# Empagliflozin targeting STAT3/Akt/Nrf2 axis promoting diabetic wound healing in rat model

**DOI:** 10.3389/fphar.2025.1678552

**Published:** 2025-09-08

**Authors:** Rania Magadmi, Dalal Alfawaz, Ahmed Bakhshwin, Fatmah Binzomah Alghamdi, Sultan A. Alfawaz, Duaa M. Bakhshwin, Maha H. Jamal, Roba A. Sofi, Ahmed Esmat

**Affiliations:** ^1^ Department of Clinical Pharmacology, Faculty of Medicine, King Abdulaziz University, Jeddah, Saudi Arabia; ^2^ Department of Pathology, Faculty of Medicine, King Abdulaziz University, Jeddah, Saudi Arabia; ^3^ Department of Clinical Physiology, Faculty of Medicine, King Abdulaziz University, Jeddah, Saudi Arabia; ^4^ Department of Pharmacology and Toxicology, Faculty of Pharmacy, Ain Shams University, Cairo, Egypt

**Keywords:** diabetes mellitus, Empagliflozin, inflammation, sodium-glucose cotransporter 2 inhibitor, wound healing

## Abstract

**Background:**

Diabetes mellitus (DM) and its complications pose a major global health challenge, with impaired wound healing leading to severe outcomes. Chronic inflammation, excessive proinflammatory cells, and high reactive oxygen species contribute to diabetic wound complications. Empagliflozin, a sodium-glucose cotransporter 2 (SGLT2) inhibitor, has antioxidant and anti-inflammatory properties, but its impact on wound healing remains unclear.

**Objective:**

To investigate the effects of empagliflozin on wound healing in diabetic rats and explore its underlying molecular mechanisms.

**Methodology:**

Fifty male Wistar rats were divided into five groups: untreated diabetic rats (STZ group), STZ + plain hydrogel, STZ + empagliflozin hydrogel (1%), STZ + oral empagliflozin (20 mg/kg), and STZ + MEBO^®^. Wounds were created 2 weeks post-STZ injection and treated for 21 days. Assessments included wound contraction, histopathology, fasting blood glucose (FBG), oxidative stress parameters, and inflammatory biomarkers in skin homogenates. Mechanistic markers, including phosphorylated STAT3, Akt, nuclear factor erythroid 2-related factor 2 (Nrf2), sirtuin 1 (SIRT1), and β-catenin, were analyzed.

**Results:**

Empagliflozin-treated animals had significant wound healing improvements, confirmed by macroscopic and histological assessments, with oral administration being the most effective. Inflammatory markers, including tumor necrosis factor-alpha (TNF- α) and interleukin-6 (IL-6), were markedly reduced, alongside decreased oxidative stress. Both oral and topical empagliflozin significantly upregulated key proteins involved in healing, including phosphorylated STAT3, Akt, Nrf2, SIRT1, and β-catenin.

**Conclusion:**

Empagliflozin accelerates wound healing in diabetic rats through its anti-inflammatory and antioxidant properties. Oral empagliflozin exhibited superior efficacy, suggesting systemic effects that extend beyond glycemic control. These findings offer insights into its molecular mechanisms of empagliflozin as a promising therapeutic agent for diabetic wound management.

## 1 Introduction

Diabetes mellitus (DM) is a debilitating and chronic condition characterized by sustained hyperglycemia. It arises from either insufficient endogenous insulin production or impaired cellular responsiveness to insulin, thereby disrupting glucose homeostasis. Irrespective of geographic region, age cohort, or gender, diabetes is universally acknowledged as a critical determinant of both global morbidity and mortality. It exerts a profound impact across diverse demographic strata (Hossain et al., 2024). As reported by the International Diabetes Federation, the global prevalence of diabetes was approximately 589 million individuals in 2024. Projections indicate a significant escalation to 852.5 million by 2050. In Saudi Arabia, an estimated 5.3 million adults aged 20–79 years were living with diabetes in 2024. This reflects a prevalence rate of 23.1% within this demographic group (International Diabetes Federation, 2025).

Mortality among individuals with diabetes is predominantly attributed to the chronic complications stemming from prolonged and severe hyperglycemia. Among these complications, non-healing wounds—particularly those affecting the lower extremities—are a notable concern (Ji et al., 2022). Impaired healing of diabetic wounds is estimated to impact nearly 25% of individuals with DM, frequently culminating in lower limb amputations. This outcome imposes substantial economic burdens and profound psychosocial ramifications on affected individuals and healthcare systems (Burgess et al., 2021). One significant manifestation of impaired wound healing is the diabetic foot ulcer (DFU), which is estimated to impact 15% of diabetic patients and contribute to nearly 84% of lower-limb amputations, highlighting their profound influence on the burden of diabetes-related complications (Memarpour et al., 2024). Several factors contribute to delayed wound healing in diabetes, including chronic hyperglycemia, which impairs vascular integrity and disrupts adequate blood perfusion. Additionally, the prevalence of peripheral vascular disease and neuropathy in diabetic patients further exacerbates the issue, often delaying wound detection and intervention (Dasari et al., 2021).

Emerging evidence highlights the potential of antidiabetic agents, such as sodium-glucose cotransporter 2 (SGLT2) inhibitors, in modulating inflammation and oxidative stress, key contributors to delayed wound healing (Miceli et al., 2024; Salazar et al., 2016; Spampinato et al., 2020). Among SGLT2 inhibitors, empagliflozin is widely utilized as an antidiabetic agent. Emerging evidence suggests that, in addition to its efficacy in glycemic regulation, empagliflozin has demonstrated pleiotropic actions, including anti-inflammatory, antioxidant, and neuroprotective effects, underscoring its potential multifaceted therapeutic benefits (Heimke et al., 2022). Recent studies suggest that empagliflozin may improve wound healing by modulating key molecular pathways such as STAT3/the nuclear factor erythroid 2-related factor 2 (Nrf2) axis, which plays a critical role in reducing oxidative stress and promoting tissue repair (Andreadou et al., 2017; Kabel et al., 2020).

Despite the promising implications, a significant gap in knowledge remains regarding the impact of empagliflozin on wound healing. Notably, the application of SGLT2 inhibitors in wound healing treatment presents a potential avenue for future clinical research. However, the therapeutic efficacy of empagliflozin in promoting wound repair is still insufficiently explored. Further investigation is needed. Therefore, this study aims to evaluate the therapeutic potential of empagliflozin in facilitating wound healing in a diabetic rat model using adult male Wistar rats, and to elucidate its underlying molecular mechanisms.

## 2 Materials and methods

### 2.1 Chemicals and reagents

Empagliflozin (SAJA Pharmaceutical Company, Jeddah, Saudi Arabia), MEBO^®^ ointment (Alnahdi Pharmacy, Jeddah, Saudi Arabia), Streptozotocin (STZ, Cat. #: S0130-1G, Merck^®^, St. Louis, MO, United States), Sodium citrate dihydrate (Merck^®^, St. Louis, MO, United States), Citric acid (Merck^®^, St. Louis, MO, United States), Dextrose (Cat. #: PHR1000, Merck^®^, St. Louis, MO, United States), Ketamine (King Abdulaziz University Hospital, Jeddah, Saudi Arabia), Xylazine (Merck^®^, St. Louis, MO, United States), Lidocaine hydrochloride 2% and Epinephrine 1:100,000 (Cook-Waite Laboratories, NY, United States), Hydroxypropyl methylcellulose (HPMC, CAS: 9004-65-3, Merck^®^, St. Louis, MO, United States), Dimethyl sulfoxide (DMSO, CAS: 67-68-5, Merck^®^, St. Louis, MO, United States), Sodium dihydrogen phosphate dihydrate, Sodium dihydrogen phosphate anhydrous, Sodium monohydrogen phosphate, and Formaldehyde solution (BDH Chemicals, Leicestershire, UK), Sodium hydroxide (NaOH, Merck^®^, St. Louis, MO, United States), Ethanol 70% (Merck^®^, St. Louis, MO, United States). All chemicals and reagents were of analytical grade.

### 2.2 Preparation of plain hydrogels, empagliflozin hydrogel and oral empagliflozin

A plain hydrogel composed solely of HPMC was formulated by adhering to the methodology outlined in an earlier research protocol. A hydrogel formulation was developed utilizing HPMC at a concentration of 2% w/v. The specified quantity of dry HPMC was precisely weighed and subsequently incorporated into half the required volume of PBS (PBS; 10 mM, pH 7.4). The mixture was vigorously stirred for 10 min to achieve uniform dispersion, after which the remaining PBS, adjusted to room temperature, was gradually incorporated to attain the target concentration. The preparation was further mixed for 10 min at room temperature using a stirrer and then subjected to a 10-min cooling phase using an ice bath. Following preparation, the storage phase involved placing the gels at 4 °C for a minimum of 48 h, eliminating any trapped air bubbles and ensuring thorough hydration of the polymers (Pan et al., 2023).

**FIGURE 1:**
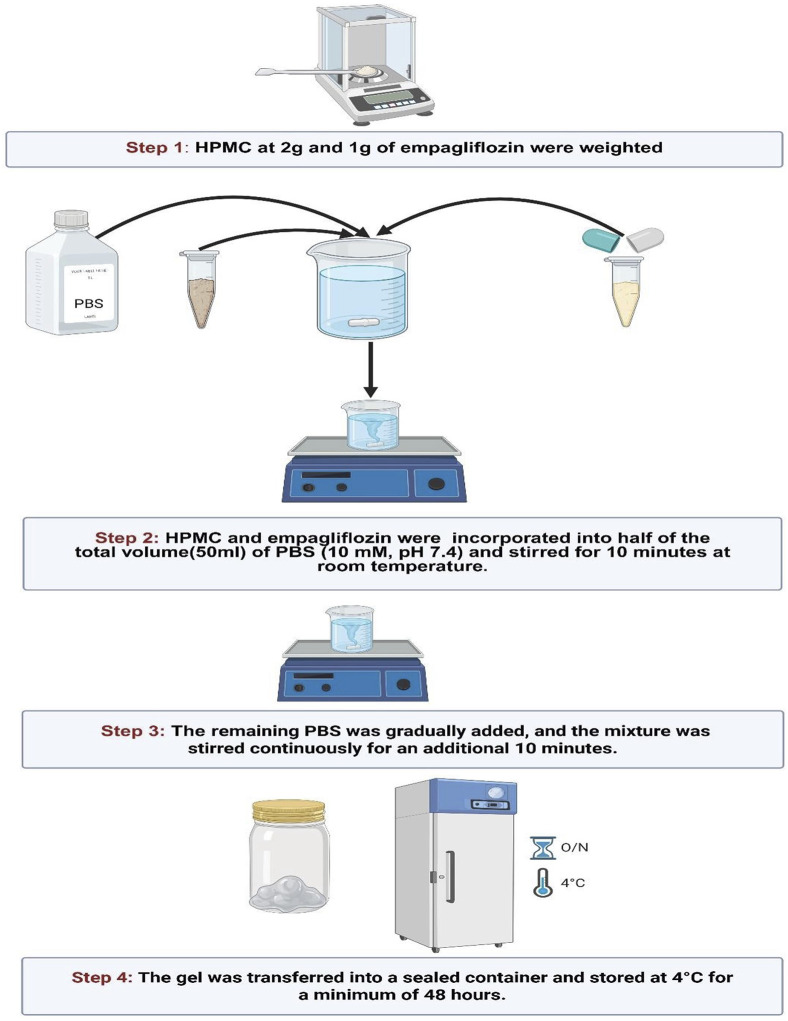
Preparation of empagliflozin hydrogel. HPMC, hydroxypropyl methylcellulose; PBS, phosphate buffered saline.

In parallel to the previous plain gel preparation, empagliflozin hydrogel was prepared as 1% w/v (Abbas et al., 2024; Pan et al., 2023; Samineni et al., 2023) by dispersing 1 g empagliflozin and 2 g HPMC in 100 mL PBS (10 mM, pH 7.4) while blending on a magnetic stirrer to ensure proper integration. After preparation, the gels were stored at 4 °C for at least 48 h to facilitate complete polymer hydration and to allow for the removal of any entrapped air bubbles, as shown in Figure 1.

The preparation of the oral empagliflozin solution began with precisely measuring 0.06 g of empagliflozin, which was subsequently dissolved in 5% DMSO/water to achieve complete solubility. The solution was thoroughly mixed using a vortex mixer to ensure uniform distribution and homogeneity. Subsequently, distilled water was gradually added while maintaining continuous mixing to achieve the desired concentration. The final solution was prepared for oral administration and administered to rats via oral gavage at a dose of 20 mg/kg/day for 21 days duration (Ekici et al., 2022).

### 2.3 Experimental design

The experimental animal study was conducted utilizing the laboratories at the Faculty of Pharmacy and the Clinical Pharmacology department at Faculty of Medicine, King Abdulaziz University, Jeddah, Saudi Arabia. Evaluation and approval of the study protocol were granted by the “Research Ethics Committee” at the Faculty of Pharmacy, King Abdulaziz University (PH-1445-32), ensuring the complete adherence to international ethical standards. The animal study was carried out over 5 weeks on 60 male Wistar rats aged 6 weeks, with an average weight between 200 and 250 g, all of whom were acquired from the animal house colony at the Faculty of Pharmacy at King Abdulaziz University. They were distributed in groups of five in their respective cages, as they were made to acclimate for 7 days preceding the initiation of the study. After which, the lighting, room temperature, and humidity were made to be consistent throughout the study duration; this included a regular 12-h light–dark cycle, an average room temperature of 23 °C–25 °C, and an average humidity of 55% ± 10%. Regarding dietary intake, an *ad libitum* standard laboratory diet was ensured, along with availability of tap water; the distribution of macronutrients was roughly as follows: 20% protein, 4% fat, and 5% fiber, among other nutrients essential for sustenance and survival.

#### 2.3.1 Induction of diabetes

Sixty male Wistar rats with a body weight ranging from 200 to 250 g were initially utilized to conduct the study. An animal model utilizing DM in rats was conducted guided by the procedure described by (Furman, 2021) with small alterations. A freshly prepared citrate buffer with a pH of 4.5 was used to dissolve STZ at a concentration of 40 mg/mL. After an overnight fast, all rats were subjected to rapid intraperitoneal injections of 40 mg/kg of STZ, which were ensured to be done under 10 minutes to avoid potential degradation of STZ within the buffer solution. All rats received a 5% dextrose solution in drinking water during the 24 h and for 1 week post diabetic induction following to minimize mortality due to STZ-induced hypoglycemia and hyperinsulinemia. Contour-next^®^, a glucometer, was utilized in the assessment of fasting blood glucose (FBG) levels and confirmation of DM 72 h post-injection—250 mg/dL was used as the cutoff for diagnosing of DM and subsequent inclusion.

#### 2.3.2 Induction of an excisional skin wound

Two weeks after the induction of diabetes, wound creation was performed (day Zero). Ketamine (80 mg/kg) along with xylazine (8 mg/kg) were utilized in anesthetizing the rats via intraperitoneal administration (Alsareii et al., 2023). In addition, 70% ethanol was utilized in the disinfection process of the shaved skin at the dorsal surface. The wound area was demarcated on the dorsal surface of the animals using a circular stencil made of stainless steel, following which a surgical excision consisting of the epidermal, dermal, and subcutaneous layers was carried out around a circular area of 1.5 cm diameter. The procedure was performed under sterile and aseptic conditions. Following thorough cleaning of the wound area, local analgesia was provided in the form of lidocaine hydrochloride (2%) and 1:100,000 epinephrine injections at the proximity of the excision area. Isolation of each rat in solitary cages was ensured afterwards, along with unrestricted access to provisions (Ghashghaii et al., 2017).

#### 2.3.3 Treatment protocol

The first group served as the control group, consisting of diabetic rats that received only STZ without any additional treatment. The second group was treated with plain hydrogel applied topically once daily. In the third group, the rats were treated with a 1% empagliflozin hydrogel, also applied topically once daily. The fourth group received oral empagliflozin at a dose of 20 mg/kg/day, administered daily. Finally, the fifth group was treated with MEBO^®^ ointment, applied topically once daily, and served as a positive control. These distinct groups facilitated a comparative analysis of the efficacy of topical and oral treatments on diabetic wound healing.

All the groups received adequate hydration in a 5-week period while drinking water consisting of 5% dextrose for 1 week post diabetic induction to avoid initial hypoglycemia. Treatments were initiated at day Zero—whereby groups 2, 3, 4, and five received plain hydrogel, empagliflozin hydrogel, oral empagliflozin, and MEBO^®^, respectively, for the duration of 21 days. The untreated STZ group received the oral vehicle 5% DMSO in water. Empagliflozin was administered orally at a dose of 20 mg/kg/day for a duration of 21 days. The dosage regimen was determined based on findings from previously conducted studies in diabetic and non-diabetic models, ensuring its appropriateness for the experimental design and therapeutic relevance (Botros et al., 2022; Ekici et al., 2022; Yaribeygi et al., 2023). Wounds were photographed on days 0, 10, and 21 and wound contraction percentage were assessed based on day Zero and 21.

**FIGURE 2:**
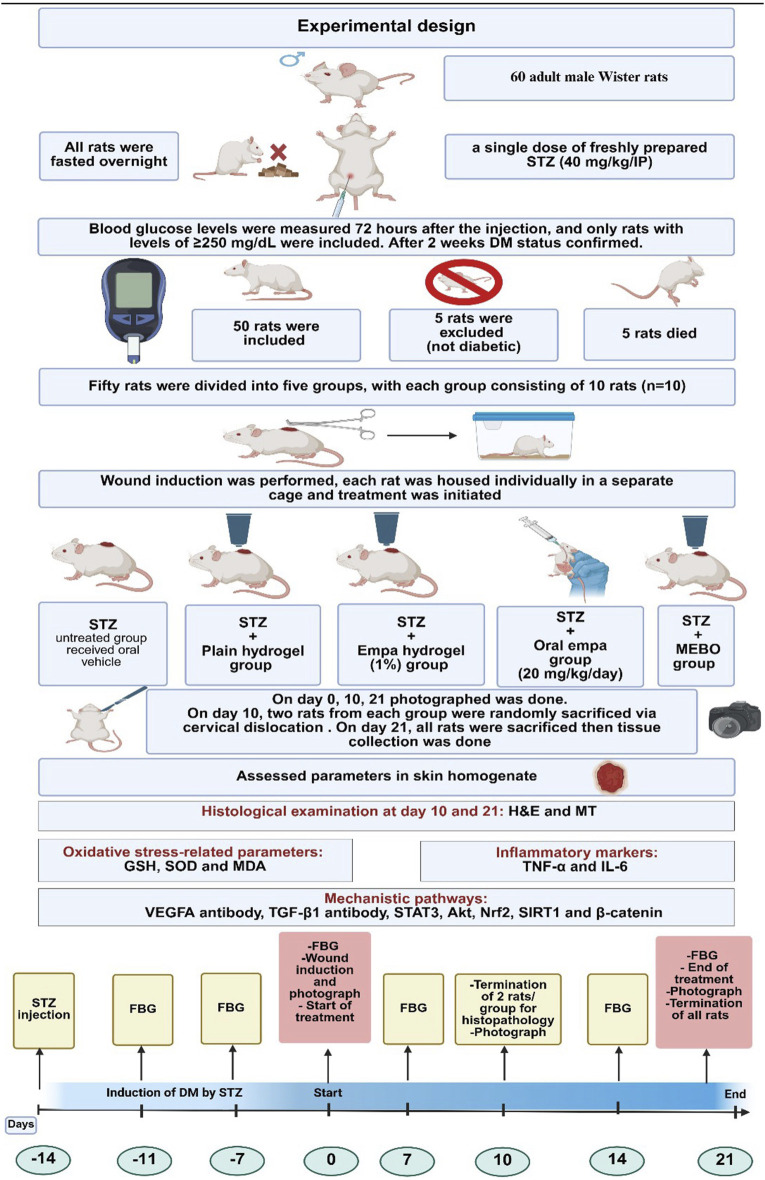
The experimental design. STZ, streptozotocin; IP, intraperitoneal; empa, empagliflozin; FBG, fasting blood glucose; GSH, reduced glutathione; SOD, superoxide dismutase; MDA, malondialdehyde; TNF-α, tumor necrosis factor-alpha; IL-6, interleukin-6; H&E, Hematoxylin and Eosin; MT, Masson’s Trichrome; VEGFA, vascular endothelial growth factor A; TGF-β1, transforming growth factor beta 1; STAT3, signal transducer and activator of transcription 3; Akt, protein kinase B; Nrf2, nuclear factor erythroid 2- related factor 2; SIRT1, sirtuin 1.

On day 10, two rats from each group were randomly anesthetized by ketamine (80 mg/kg)/xylazine (8 mg/kg) via intraperitoneal administration (Alsareii et al., 2023), then sacrificed via cervical dislocation to assess the early effect of medication. Thereafter, skin from the wound area was dissected, and the tissue was preserved in 10% neutral formalin. On day 21, all rats were anesthetized -as previously mentioned - then sacrificed, and representative skin tissues from 2 rats/group were kept in 10% neutral formalin for further histopathological evaluation. All remaining tissues were flash-frozen in liquid nitrogen and stored at −80 °C for subsequent biochemical analyses. The experimental design is summarized in Figure 2.

#### 2.3.4 Wound contraction rate

The extent of wound contraction was calculated using the formula outlined below:

$$Wound\;contraction\;\left(\%\right)=\frac{\text{Day}\;\text{Zero}\;\text{wound}\;\text{diameter}-\text{Day}\;21\;\text{wound}\;\text{diameter}}{\text{Day}\;\text{Zero}\;\text{wound}\;\text{diameter}}$$

#### 2.3.5 Assessment of molecular markers

The parameters evaluated in this study were categorized into diabetic markers, oxidative stress-related parameters, inflammatory markers, and mechanistic pathway markers. Fasting blood glucose (FBG) was measured to assess glycemic status. Oxidative stress-related parameters included reduced glutathione (GSH; Cat. #: GR 25-11), superoxide dismutase (SOD; Cat. #: SD 25-21), and malondialdehyde (MDA; Cat. #: MD 25-29), with all kits purchased from Bio-Diagnostic (Giza, Egypt). Inflammatory markers, including tumor necrosis factor-alpha (TNF-α; Cat. #: SEA133ra) and interleukin-6 (IL-6; Cat. #: SEA079ra), were quantified using ELISA kits obtained from Cloud-Clone Corp. (Texas, United States). Mechanistic pathway markers assessed included signal transducer and activator of transcription 3 (STAT3; Cat. #: MBS1608710), protein kinase B (Akt; Cat. #: MBS9511022), Nrf2 (Cat. #: MBS012148), SIRT1 (Cat. #: MBS2515967), and β-catenin (Cat. #: MBS1607577), with all assay kits purchased from MyBioSource (San Diego, CA, United States). All assays were performed according to the manufacturers’ instructions to ensure accuracy and reproducibility. Nrf2 expression was assessed in the nuclear fraction of the tissue homogenate using NE-PER nuclear and cytoplasmic extraction kit (Cat. #: 78833, Thermo Scientific, IL, United States) for preparation of nuclear fractions. The nuclear protein content was then determined, and an equal amount of nuclear protein was employed in the assessment of the concentration of Nrf2.

### 2.4 Statistical analysis

The results are presented as the mean ± standard deviation (SD), based on eight replicates (n = 8). All parameters were statistically evaluated using one-way ANOVA, followed by Tukey’s post-hoc test to conduct pairwise comparisons. Repeated measures ANOVA was specifically utilized to analyze FBG levels. Statistical comparisons were conducted as follows: “a” denotes a statistically significant difference relative to the STZ group, “b” indicates a statistically significant difference relative to the STZ + plain hydrogel group, “c” represents a statistically significant difference relative to the STZ + empagliflozin hydrogel group, and “d” signifies a statistically significant difference relative to the STZ + oral empagliflozin group. A significance threshold of *P <* 0.05 was applied to determine statistical relevance. All statistical analyses and graph generation were carried out using GraphPad Prism^®^ version 8 (GraphPad Software, LLC, CA, United States).

## 3 Results

### 3.1 Empagliflozin promoted wound healing in diabetic rats

**FIGURE 3:**
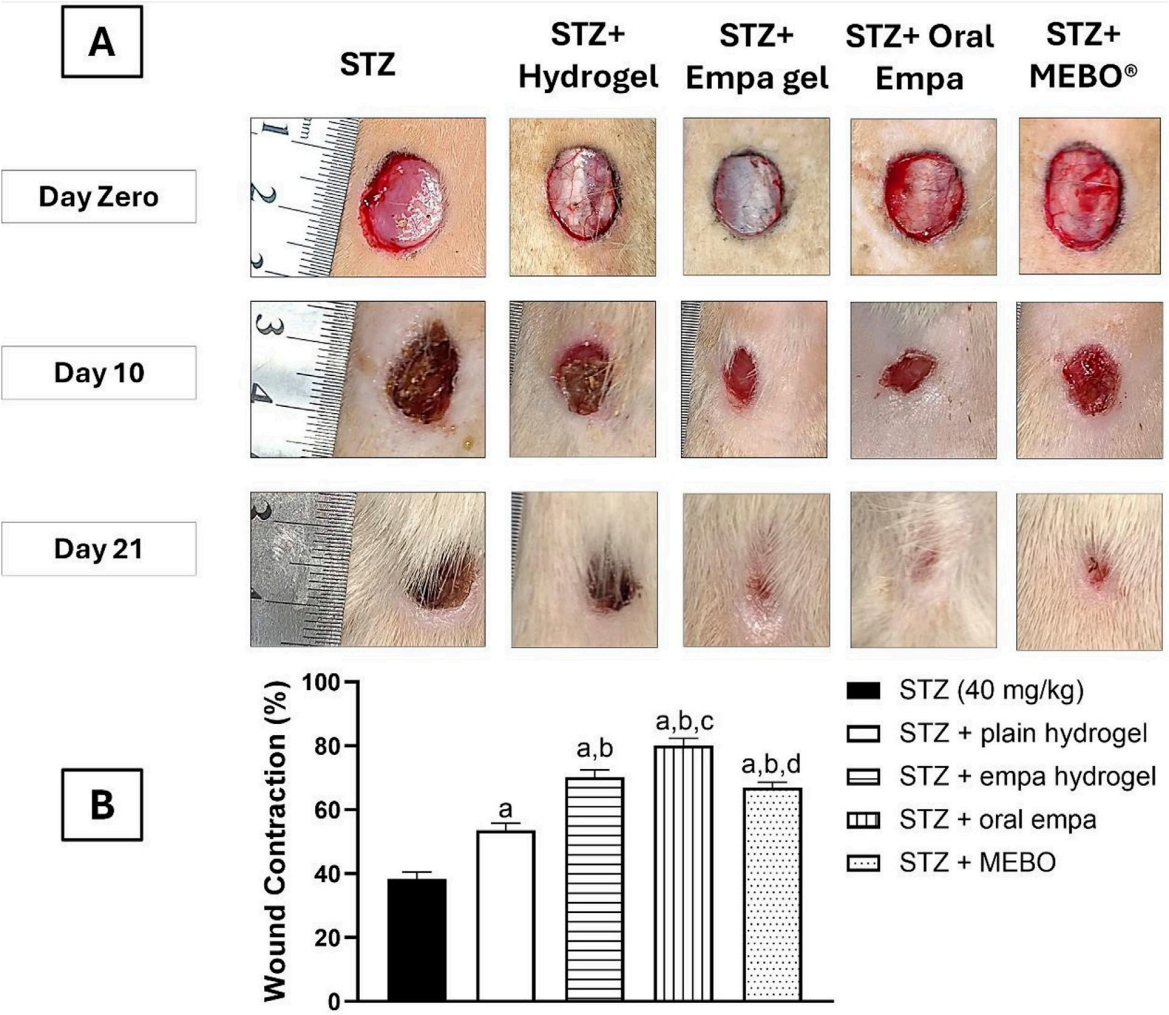
The Effect of Empagliflozin on the wound closure **(A)** and wound contraction percentage on day 21 **(B)**. Data are displayed as mean ± SD. n = 8. Statistical analysis was performed by one-way ANOVA followed by Tukey’s post-hoc test. a: Statistically significant from the STZ group at *P <* 0.05. b: Statistically significant from the STZ + plain hydrogel treated group at *P <* 0.05. c: Statistically significant from the STZ + empagliflozin hydrogel (1%) treated group at *P <* 0.05. d: Statistically significant from the STZ + oral empagliflozin (20 mg/kg) treated group at *P <* 0.05. STZ, streptozotocin; empa, empagliflozin.

As shown in Figure 3A, the wound size demonstrated a gradual reduction following treatment with empagliflozin hydrogel and oral empagliflozin at day 10 and 21 following treatment. The percentage of wound contraction was calculated as in Figure 3B in which treatment with empagliflozin hydrogel, oral empagliflozin, and MEBO^®^ significantly accelerated wound healing (*P <* 0.05) by 70.18%, 80.24%, and 67%, respectively, compared to animals received STZ alone, which showed an improvement of only 38.41%.

Interestingly, plain hydrogel alone significantly enhanced wound healing contraction (*P <* 0.05), achieving an improvement of 53.65% compared to the STZ group, which exhibited only 38.41% healing. However, the application of empagliflozin hydrogel, oral empagliflozin, and MEBO^®^ resulted in significantly accelerated wound healing (*P <* 0.05) by day 21 compared to plain hydrogel.

By day 21, there was no statistically significant difference observed between wounds treated with MEBO^®^ and those treated with empagliflozin hydrogel. Notably, wounds exposed to oral empagliflozin exhibited the most significant wound contraction (*P <* 0.05) by day 21, achieving 80.24% contraction. This was higher than that observed with empagliflozin hydrogel and MEBO^®^, indicating that animals receiving oral empagliflozin demonstrated the greatest healing efficacy.

### 3.2 Histological analysis of empagliflozin treatments

**FIGURE 4:**
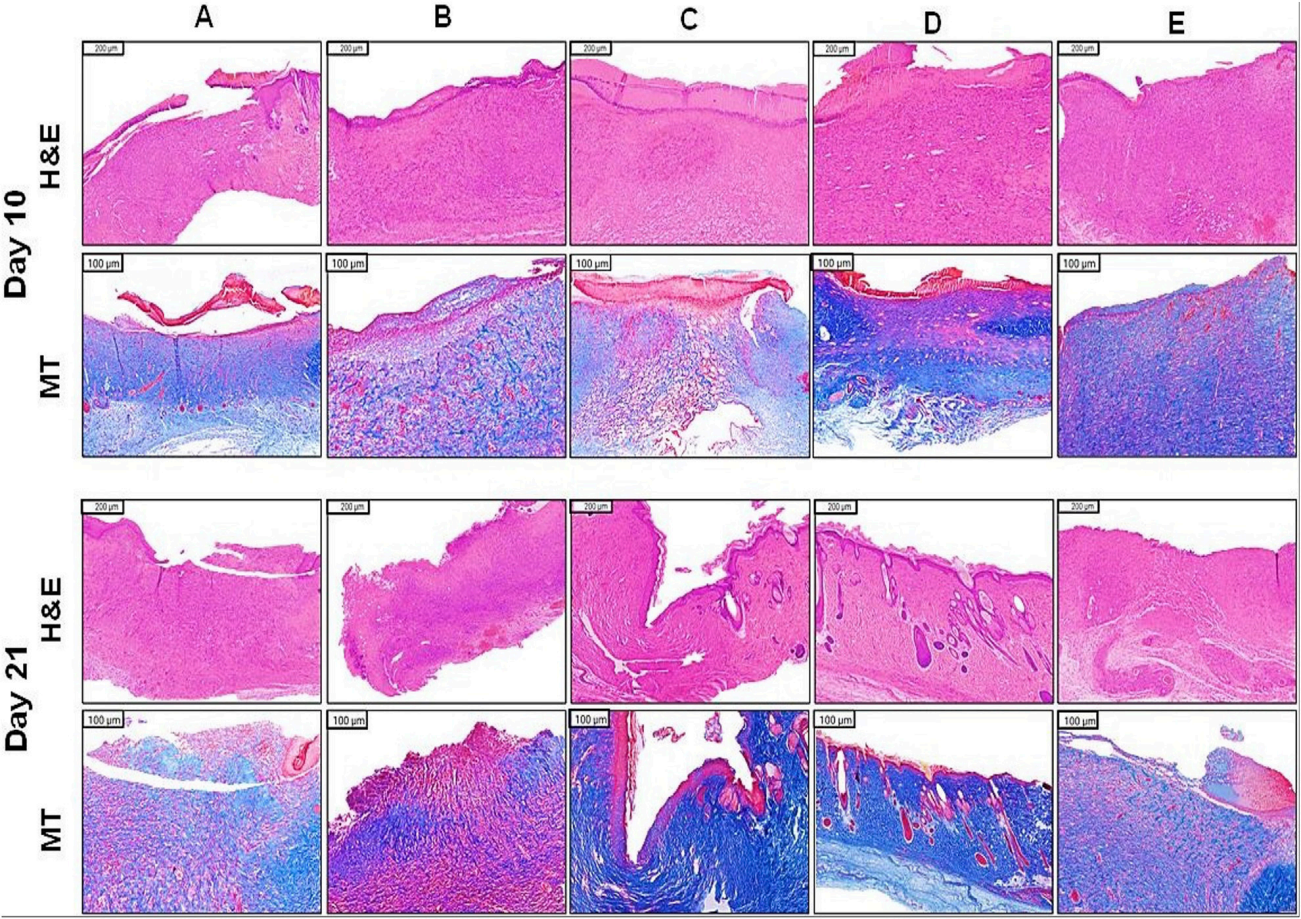
Histopathological examination in wounded skin of diabetic rats. **(A)** STZ (40 mg/kg), **(B)** STZ + plain hydrogel, **(C)** STZ + empa hydrogel (1%), **(D)** STZ + oral empa (20 mg/kg), **(E)** STZ + MEBO^®^. Skin sections from the wounded areas were collected on days 10 and 21 and subsequently stained with H&E and MT to evaluate tissue morphology and collagen deposition. STZ, streptozotocin; H&E, hematoxylin and eosin; MT, masson’s trichrome; empa, empagliflozin.

The histopathological examination, as depicted in Figure 4, reveals clear patterns of wound healing among the treatment groups on Days 10 and 21. The STZ group consistently exhibited minimal progress in wound healing. On day 10, the histological examination revealed sparse collagen fibers (MT staining), reduced fibroblast activity, and poorly developed granulation tissue. By day 21, slight improvements were evident; however, collagen organization and overall tissue regeneration remained insufficient and suboptimal.

Similarly, the STZ + plain hydrogel group demonstrated only marginal improvements compared to the STZ group. On day 10, collagen deposition (MT staining) and granulation tissue formation were minimal, while by day 21, slight increases in fibroblast activity and vascularization were observed. Nevertheless, overall wound healing outcomes remained subpar relative to other treated groups.

In contrast, the STZ + empagliflozin hydrogel group exhibited moderate wound healing progress by day 10, characterized by moderate epidermal regeneration and initial granulation tissue development. By day 21, significant advancements were observed, including improved collagen organization (MT staining), heightened fibroblast activity, and well-developed granulation tissue, underscoring the therapeutic potential of this treatment approach.

The STZ + oral empagliflozin group consistently outperformed all other treatments. By day 10, significant enhancements in fibroblast activity, collagen deposition (MT staining), and vascularization were observed. By day 21, this group exhibited the most advanced healing outcomes, characterized by complete epidermal regeneration, highly organized collagen (MT staining), and fully matured granulation tissue. These findings collectively highlight the superior efficacy of empagliflozin-based treatments, particularly oral empagliflozin, in outperforming MEBO^®^, plain hydrogel, and the untreated control group in promoting wound healing.

The STZ + MEBO^®^ group displayed consistent progress in wound healing. By day 10, minimal epidermal regeneration and fibroblast activity were noted, accompanied by early granulation tissue formation. By day 21, substantial improvements in collagen organization (MT staining) and the maturation of granulation tissue were apparent, though the overall outcomes were slightly less effective compared to those observed with empagliflozin-based treatments.

Table 1 indicates the findings of semi-quantitative evaluation of the histological examination at day 21 (Celani et al., 2019; Durmaz et al., 2012). As shown in the table, diabetic rats treated with oral empagliflozin (20 mg/kg) displayed the highest rate of healing in terms of epidermal regeneration, collagen fiber formation, fibroblast count, and vascularity.

**TABLE 1:** Semi-quantitative characterization of wounded skin of diabetic rats on day 21.

Groups	ER	CF	F	V	RE	GTF	CO
STZ (40 mg/kg)	+	-	+	+	+	++	++
STZ + plain hydrogel	+	+	+	+	+ + +	+ + +	+ +
STZ + empa hydrogel (1%)	+ +	+ +	+ +	+	+ + + +	+ + + +	+ + + +
STZ + oral empa (20 mg/kg)	+ + +	+ + +	+ + +	++	+ + + +	+ + + +	+ + + +
STZ + MEBO^®^	+	+ +	+	+	+ + +	+ + +	+ +

The symbols -, +, ++, +++ and ++++ represent the following levels: none, low, moderate, high, and extreme; respectively.

STZ, streptozotocin; empa, empagliflozin; ER, epidermal regeneration; CF, collagen fibers; F, numbers of fibroblasts; V, vascularity; RE, re-epithelialization; GTF, granulation tissue formation; CO, collagen organization.

### 3.3 Oral empagliflozin reduced fasting blood glucose levels in wounded diabetic rats

**FIGURE 5:**
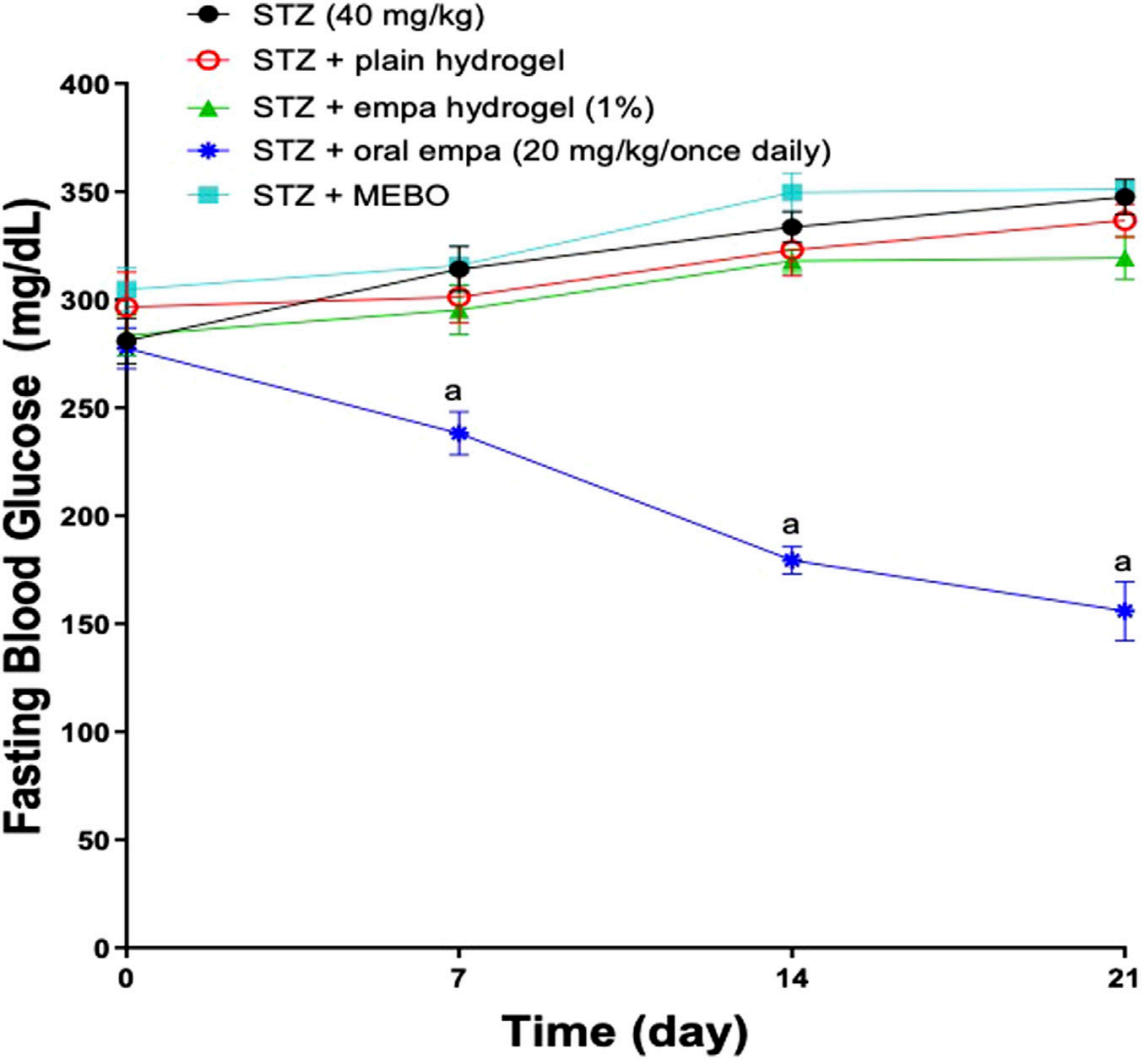
Assessment of Fasting Blood Glucose levels in wounded diabetic rats. Data are represented as mean ± S.D. n = 8. The statistical analysis was conducted using repeated measures ANOVA, followed by Tukey’s post-hoc test. a: Statistically significant from the STZ group at *P <* 0.05. STZ, streptozotocin; FBG, fasting blood glucose; empa, empagliflozin.

Figure 5 illustrates the FBG levels observed in different groups throughout the 5-week experimental period. Prior to the STZ injection (day −14), no notable differences were observed among the groups compared to the STZ group. One week after the STZ injection (day −7) and 2 weeks after the injection (day Zero), FBG levels showed a significant increase (*P <* 0.05) in all groups. All treatments were initiated on day Zero.

Notably, treatment with oral empagliflozin (20 mg/kg) resulted in a significant reduction (*P <* 0.05) in FBG levels, by 24% related to the STZ group by day 7. This decline in FBG levels continued progressively until the end of the study. By day 21 of the experiment, animals treated orally with empagliflozin (20 mg/kg) demonstrated a significant reduction (P < 0.05) in FBG levels, decreasing to 55% of the levels observed in the STZ group.

Remarkably, oral administration of empagliflozin (20 mg/kg) demonstrated significantly greater efficacy in reducing FBG levels compared to both plain hydrogel and empagliflozin hydrogel, as well as MEBO^®^ treatments, which exhibited no significant impact on FBG reduction. This effect was observed on day 7, which corresponds to 1 week following the initiation of treatment and continued until day 21 of the experiment.

### 3.4 Empagliflozin improved oxidative stress-related parameters

#### 3.4.1 Content of reduced glutathione in skin homogenate

**FIGURE 6:**
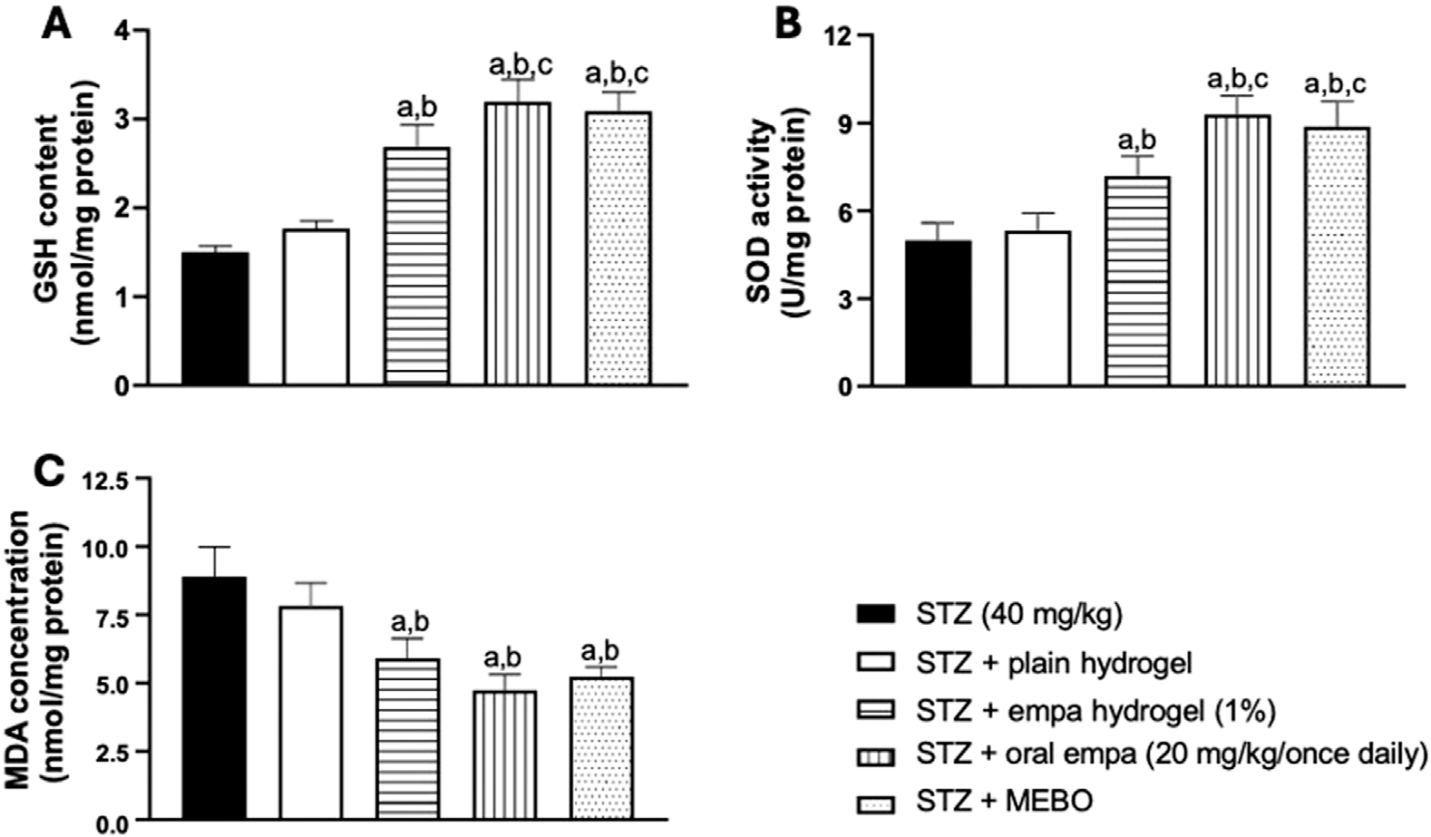
Effect on oxidative stress-related parameters **(A)** glutathione (GSH), **(B)** superoxide dismutase (SOD), and **(C)** malondialdehyde (MDA) in wounded skin of diabetic rats. The data are expressed as mean ± SD. n = 8. Statistical analysis was carried out by one-way ANOVA followed by Tukey’s post-hoc test. a: Statistically significant from the STZ group at *P* < 0.05. b: Statistically significant from the STZ + plain hydrogel treated group at *P* < 0.05. c: Statistically significant from the STZ + empagliflozin hydrogel (1%) treated group at *P* < 0.05. STZ, streptozotocin; empa, empagliflozin.

As presented in Figure 6A, animals treated with oral empagliflozin and MEBO^®^ demonstrated significant (*P <* 0.05) increases in GSH content, exceeding a one-fold elevation compared to the STZ group. On other side, treatment with empagliflozin hydrogel resulted in a significant (*P <* 0.05) enhancement in GSH content, by 79% compared to the STZ group. However, the GSH content in the STZ + plain hydrogel group was not significantly different from that of the STZ group.

Furthermore, both oral empagliflozin and MEBO^®^ groups showed a statistically significant difference (*P <* 0.05), achieving about 80% enhancement in GSH content compared to plain hydrogel. Additionally, oral empagliflozin and MEBO^®^ treatments demonstrated greater efficacy than the STZ + empagliflozin hydrogel group, with GSH content increasing by approximately 19% for oral empagliflozin and 15% for MEBO^®^. However, no significant difference was observed between the effects of oral empagliflozin and MEBO^®^ treatments.

#### 3.4.2 Activity of superoxide dismutase in skin homogenate

By evaluating the SOD enzymatic activity, no significant difference was observed between the STZ + plain hydrogel group and the STZ group, as presented in Figure 6B. On the other hand, treatments with empagliflozin hydrogel, oral empagliflozin, and MEBO^®^ demonstrated significant (*P <* 0.05) enhancements of SOD activity compared to the STZ group.

Notably, treatment with oral empagliflozin was more efficient than the gel form, being able to trigger a 29% increase in SOD activity compared to STZ + empagliflozin hydrogel group. Once again, no statistically significant difference was observed between the effects of oral empagliflozin and MEBO^®^ treatments.

#### 3.4.3 Concentration of malondialdehyde in skin homogenate

Treatment with empagliflozin hydrogel, oral empagliflozin and MEBO^®^ achieved significant reductions in MDA concentration by 34%, 47% and 41%, respectively, compared to the STZ group as shown in Figure 6C. The percentages of reductions were comparable among the three previously mentioned groups. However, plain hydrogel treatment did not yield any significant difference in MDA concentration compared to the STZ group.

### 3.5 Empagliflozin reduced inflammatory markers in diabetic wound

#### 3.5.1 Concentration of TNF- α in skin homogenate

**FIGURE 7:**
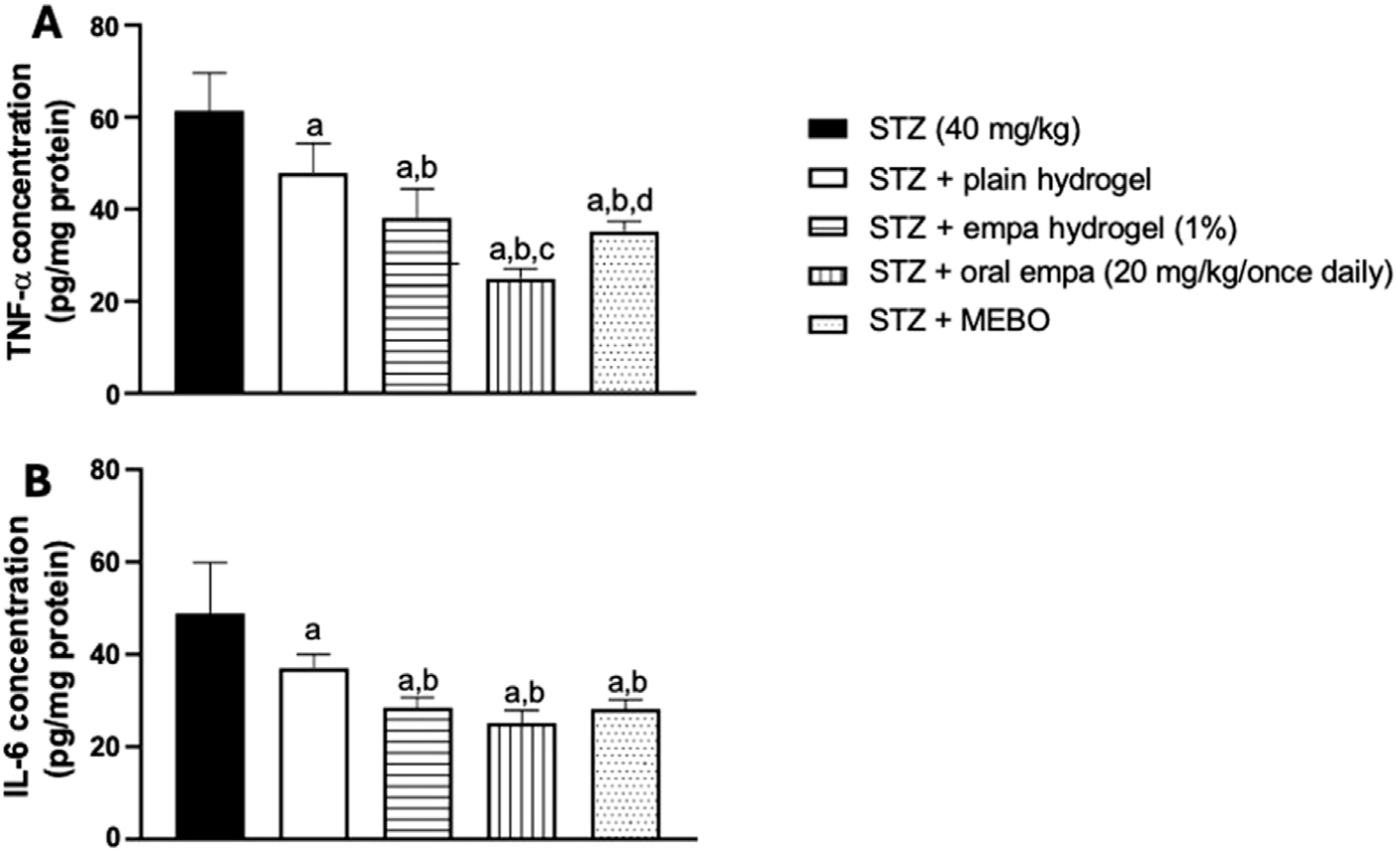
Effect on inflammatory biomarkers **(A)** tumor necrosis factor-alpha (TNF-α), and **(B)** interleukin-6 (IL-6) in wounded skin of diabetic rats. The data are expressed as mean ± S.D. n = 8. Statistical analysis was performed by one-way ANOVA followed by Tukey’s post-hoc test. a: Statistically significant from the STZ group at *P* < 0.05. b: Statistically significant from the STZ + plain hydrogel treated group at *P* < 0.05. c: Statistically significant from the STZ + empagliflozin hydrogel (1%) treated group at *P* < 0.05. d: Statistically significant from the STZ + oral empagliflozin (20mg/kg) treated group at *P* < 0.05. STZ, streptozotocin; empa, empagliflozin.

As illustrated in Figure 7A, all treatment groups showed anti- inflammatory activities. The plain hydrogel, empagliflozin hydrogel, oral empagliflozin, and MEBO^®^ groups demonstrated a significant reduction (*P <* 0.05) in TNF-α concentration by 22%, 38%, 59%, and 43%, respectively, compared to the STZ group.

Empagliflozin hydrogel and MEBO^®^ treatments were capable of significantly reducing TNF-α concentration by 20% and 26%, respectively, compared to plain hydrogel. Furthermore, oral empagliflozin exhibited the most pronounced effect, achieving a 48% reduction in TNF-α concentration compared to plain hydrogel. These differences were statistically significant, highlighting the superior anti-inflammatory potential of oral empagliflozin and topical treatments over plain hydrogel.

When comparing oral and topical empagliflozin, oral treatment showed a significant (*P <* 0.05) reduction in TNF-α concentration by approximately 34% compared to the topical- treated group.

#### 3.5.2 Concentration of interleukin-6 in skin homogenate

In a similar pattern, significant (*P <* 0.05) reductions in IL-6 concentration were detected across the plain hydrogel, empagliflozin hydrogel, oral empagliflozin, and MEBO^®^ groups compared to the STZ group, as shown in Figure 7B. Specifically, plain hydrogel treatment led to a 24% reduction in IL-6 concentration compared to the STZ group. In contrast, empagliflozin hydrogel, oral empagliflozin, and MEBO^®^ treatments resulted in more pronounced reductions of 42%, 48%, and 42%, respectively, relative to the STZ group.

Interestingly, when comparing all treated groups to plain hydrogel, oral empagliflozin exhibited the greatest reduction in IL-6 concentration at 32%, followed by MEBO^®^ group by a 24% reduction, and empagliflozin hydrogel with a 23% reduction. However, neither empagliflozin hydrogel nor MEBO^®^ treatment produced a statistically significant difference compared to oral empagliflozin.

### 3.6 Empagliflozin activated STAT3, Akt, Nrf2, SIRT1, and β-catenin pathways in wounded skin of diabetic rats

#### 3.6.1 Concentration of phosphorylated signal transducer and activator of transcription 3 in wounded skin of diabetic rats

**FIGURE 8:**
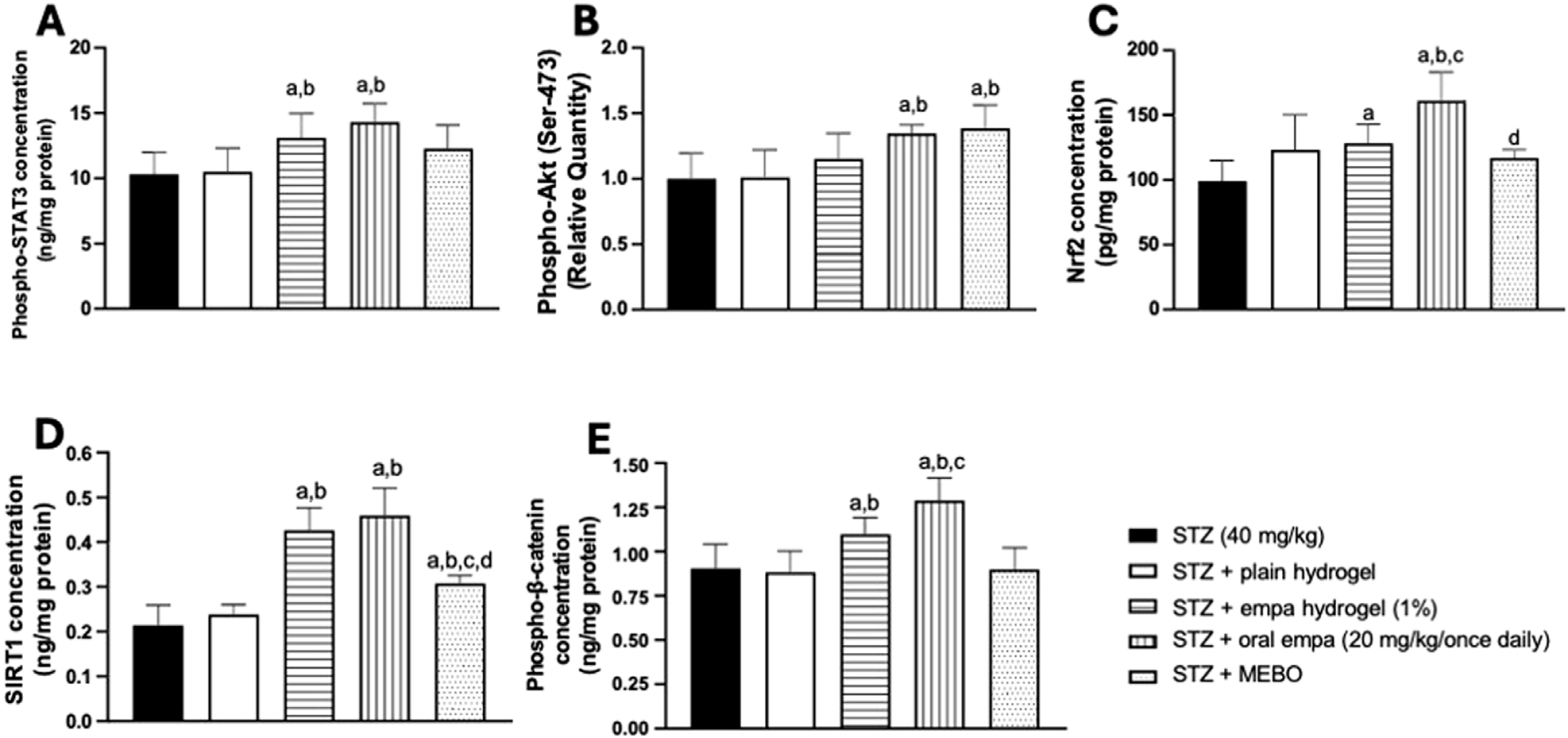
Effects on mechanistic pathways in wounded skin of diabetic rats. **(A)** phosphorylated signal transducer and activator of transcription 3 (phospho-STAT3); **(B)** protein kinase B (phospho-Akt); **(C)** nuclear factor erythroid 2- related factor 2 (Nrf2); **(D)** sirtuin 1 (SIRT1); **(E)** phosphorylated β-catenin (Phospho-β-catenin). Data are presented as mean ± S.D. n = 8. Statistical analysis was carried out by one-way ANOVA followed by Tukey’s post-hoc test. a: Statistically significant from STZ group at *P* < 0.05. b: Statistically significant from the STZ + plain hydrogel treated group at P < 0.05. c: Statistically significant from the STZ + empagliflozin hydrogel (1%) treated group at *P* < 0.05. d: Statistically significant from the STZ + oral empagliflozin (20mg/kg) treated group at *P* < 0.05. STZ, streptozotocin; empa, empagliflozin.

The activity of STAT3 was analyzed by quantifying the concentration of its phosphorylated (active) form in rat skin homogenates. As presented in Figure 8A, groups treated with topical and oral empagliflozin exhibited comparable significant increases (*P <* 0.05) in phosphorylated STAT3 concentration, by about 27% and 39%, respectively, compared to the STZ group. However, treatment with plain hydrogel and MEBO^®^ failed to show any significant difference from the STZ group.

#### 3.6.2 Relative quantity of phosphorylated Akt (Ser-473) in wounded skin of diabetic rats

As indicated in Figure 8B, treatments with oral empagliflozin and MEBO^®^ showed similar effects when directly compared to both the STZ group and the plain hydrogel group. The relative quantity of phosphorylated Akt was significantly increased (P < 0.05) by 35% and 41% in the groups treated with oral empagliflozin and MEBO^®^, respectively, compared to the STZ group. However, despite these notable improvements, the difference between oral empagliflozin and MEBO^®^ was not statistically significant.

In contrast, neither plain hydrogel nor empagliflozin hydrogel treatments induced any significant changes in the relative quantity of Akt compared to the STZ group.

#### 3.6.3 Concentration of nuclear factor erythroid 2-related factor 2 in wounded skin of diabetic rats

As presented in Figure 8C, Nrf2 nuclear concentration was significantly elevated (*P <* 0.05) in animals treated with oral empagliflozin and empagliflozin hydrogel, by approximately 29% and 62%, respectively, compared to the STZ group. Notably, oral empagliflozin demonstrated the highest effectiveness in enhancing Nrf2 nuclear concentration, contributing to improved wound healing. This oral treatment exhibited a statistically significant increase of 26% in Nrf2 nuclear concentration compared to the empagliflozin hydrogel group and 31% relative to the plain hydrogel.

Interestingly, while the MEBO®-treated group showed a marginal increase in nuclear Nrf2, it was statistically significantly lower than oral empagliflozin. In addition, no significant differences were observed between the plain hydrogel and MEBO^®^ groups when compared to the STZ group.

#### 3.6.4 Concentration of sirtuin 1 in wounded skin of diabetic rats

The data presented in Figure 8D indicate that SIRT1 concentration significantly increased (*P <* 0.05) in both the empagliflozin hydrogel and oral empagliflozin treatment groups - in a comparable manner - by about one-fold relative to the STZ group. Besides, MEBO^®^ treatment induced a significant (*P <* 0.05) increase in SIRT1 concentration by 48% compared to the STZ group. However, the effect of MEBO^®^ was much less than that of empagliflozin, whether applied topically or taken orally. Conversely, treatment with plain hydrogel failed to produce any significant change when compared to the STZ group.

#### 3.6.5 Concentration of phosphorylated β-catenin in wounded skin of diabetic rats

The functionality of β-catenin was analyzed by quantifying the phosphorylated (active) form in rat skin homogenate. The group treated with empagliflozin hydrogel demonstrated a significant increase (*P <* 0.05) by 23% compared to the STZ group (Figure 8E). Moreover, the group that received oral empagliflozin exhibited a significantly greater increase (*P <* 0.05) of 43% relative to the STZ group. Both the plain hydrogel and MEBO^®^ groups failed to produce any significant change in phosphorylated β- catenin concentration when compared to the STZ group.

It is worth highlighting that oral empagliflozin has the greatest effect on enhancing β-catenin activity, being more effective than empagliflozin hydrogel by 16%.

## 4 Discussion

One of the chronic metabolic disorders is DM, marked by persistent hyperglycemia that significantly impairs wound healing by disrupting essential reparative mechanisms and leading to prolonged and delayed recovery (Burgess et al., 2021). Moreover, alongside persistent hyperglycemia, diabetic wound healing is further complicated by elevated oxidative stress, inflammation and other molecular pathways, which hinder normal reparative processes. This exacerbates the complexity of recovery, making it a formidable challenge for those with diabetes (Astaneh and Fereydouni, 2024; Zhao J. et al., 2021). The continuation of diabetic complications following the resolution of hyperglycemia hints at a phenomenon known as metabolic memory. Yet, the influence of this metabolic memory on diabetic wound healing and the associated molecular mechanisms remains inadequately understood (Zhao J. et al., 2021). These complexities render diabetic wound treatment particularly challenging, and despite ongoing efforts, an optimal therapeutic approach remains elusive. Consequently, there is an urgent need for innovative strategies to effectively manage and enhance diabetic wound healing. This study was undertaken to investigate the potential therapeutic effects and underlying mechanistic pathways of empagliflozin, administered both topically and orally, on diabetes- induced wound in rat model.

The current study utilized a meticulously designed diabetic wounded animal model to replicate the delayed wound healing observed in diabetic patients, ensuring translational relevance to human diabetic wound repair. The experimental diabetes in rats is most effectively and efficiently achieved through the administration of STZ, a compound exhibiting selective cytotoxicity toward pancreatic β-cells (Mostafavinia et al., 2016). The deleterious effects of STZ on β-cells are predominantly mediated by its ability to induce DNA alkylation. This leads to significant alterations in systemic glucose and insulin homeostasis. Within approximately 6 hours post-administration, a transient phase of pronounced hypoglycemia occurs, distinguished by elevated circulating insulin levels. This is followed by the onset of hyperglycemia, concurrent with a substantial decline in plasma insulin concentration. These sequential perturbations underscore the profound β-cell dysfunction caused by STZ, ultimately resulting in their irreversible destruction (Szkudelski, 2001).

Empagliflozin, an antidiabetic agent, has demonstrated a profound ability to mitigate ROS, pivotal contributors to vascular damage, while simultaneously inducing autophagy—an essential cellular repair mechanism. Additionally, it exerts significant anti-inflammatory effects, further enhancing its therapeutic potential. These combined actions are intricately linked to improved wound healing processes and the amelioration of diabetic peripheral neuropathy, a critical factor in reducing the heightened risk of chronic, non-healing wounds in individuals with diabetes (Miceli et al., 2024).

Topical drug delivery is widely valued for its targeted treatment of skin disorders but often faces challenges like poor retention and limited penetration. However, hydrogels have emerged as an effective medium for controlled and sustained release, helping to overcome these limitations (Pan et al., 2023). The formulation of the hydrogel is supported by previously reported evidence highlighting its remarkable attributes as a superior class of wound dressings, esteemed for their antimicrobial efficacy, exceptional drug delivery potential, excellent biocompatibility and biodegradability, and rapid hemostatic capabilities (Yu et al., 2024). However, the inherently hydrophilic polymer matrix of hydrogels presents challenges in delivering hydrophobic drugs, requiring modifications to effectively incorporate compounds with poor water solubility (Larraneta et al., 2018). In this context, empagliflozin is a hydrophobic, characterized by its limited water solubility, and exhibits pronounced lipophilic properties, which are integral to its molecular structure and pharmacokinetic behavior (Gumieniczek et al., 2024). One of the widely used polymers in controlled-release systems is HPMC, prized for its abilities in thickening, gel formation, and swelling. These properties facilitate the production of hydrogels that are exceptionally stable, transparent, and odor-free (Ghorpade et al., 2016). Remarkably, HPMC demonstrates a unique combination of hydrophilic and hydrophobic properties (Chiaregato et al., 2023). Due to its unique structure, HPMC, possessing both hydrophilic and hydrophobic moieties, acts as a water-soluble polymeric solubilizer capable of interacting with poorly water-soluble molecules. Its alkyl ether side chains and cyclic ether rings form site-specific hydrophobic pockets, enhancing aqueous viscosity and facilitating molecular interactions (Petkova et al., 2020). Owing to its numerous advantages, HPMC was added to both plain and empagliflozin hydrogels to enhance their properties.

In the Middle East and Asia, MEBO^®^, an oil-based herbal ointment, is commonly used and is considered effective for managing burn wounds (Mabvuure et al., 2020). It contains several active ingredients, each contributing to its therapeutic efficacy. Berberine oil provides antimicrobial properties, helping to prevent infection during wound healing. Sesame oil works to alleviate pain, retain moisture, and soften the wound site, promoting a favorable environment for recovery. Beta-sitosterol, a plant-based steroid, is known to stimulate epithelialization, aiding in faster skin regeneration, while also exhibiting strong anti-inflammatory effects, as supported by numerous studies (Najim et al., 2022). The lipid-based component of MEBO^®^ is believed to enhance moisture retention within the wound environment, thereby supporting optimal healing conditions (Mabvuure et al., 2020). Research has demonstrated the efficacy of MEBO^®^ in wound healing through an *in vivo* study on diabetic mice (Gong et al., 2023). Therefore, MEBO^®^, was used in this study as a positive control for empagliflozin hydrogels.

In the present study, the effect of empagliflozin on wound healing was evaluated through a combination of macroscopic and histological analyses. Macroscopic observations were based on photographic documentation captured on days 10 and 21, providing visual insights into the progression of tissue repair. Additionally, histological assessments were conducted using H&E staining as well as MT staining at the same time points (days 10 and 21) to offer detailed cellular and structural evaluations of the healing process. The findings reveal that empagliflozin, administered via both topical and oral routes, exhibited significantly superior efficacy in promoting wound healing compared to other treatments. This was evident in both gross morphological observations and histological assessments (Figures 3, 4). Empagliflozin outperformed MEBO^®^ and plain hydrogel and was significantly more effective compared to the untreated STZ group, which demonstrated a notably slower rate of improvement. These results underscore the pronounced therapeutic potential of empagliflozin in accelerating and enhancing tissue repair processes.

Notably, oral administration of empagliflozin at a dose of 20 mg/kg/day exhibited the most significant improvement in wound healing, surpassing both topical empagliflozin hydrogel and MEBO^®^. This accelerated healing effect is likely attributable, at least in part, to its ability to lower FBG levels (Xiang et al., 2019). This suggests that its multifaceted mechanism of action involves both systemic glycemic control and additional molecular pathways independent of glucose regulation. Indeed, the topical application of empagliflozin hydrogel significantly accelerated the wound healing process more effectively than MEBO^®^, irrespective of its influence on FBG levels, as the topical application of empagliflozin demonstrated no measurable impact on FBG regulation.

In comparison to the untreated STZ group, significant improvements were observed following MEBO^®^ treatment. These findings regarding MEBO^®^ substantiate previous investigations in diabetic mice, which demonstrated that MEBO^®^ significantly enhanced the formation of granulation tissue and triggered robust collagen remodeling (Gong et al., 2023). Although MEBO^®^ demonstrated greater efficacy in wound healing compared to plain hydrogel, the plain hydrogel itself appeared to impede the progression of wound healing relative to the untreated group. This effect could be explained by the intrinsic properties of hydrogels, which maintain a moist environment, facilitate oxygen diffusion, and sustain elevated moisture levels at the wound site. Accordingly, hydrogel-based interventions have emerged as promising therapeutic agents by effectively absorbing wound exudates, adhering firmly, and exhibiting shape adaptability and mechanical protection, thereby expediting the healing process (Gounden and Singh, 2024).

It is imperative to acknowledge that a significant phenomenon influencing human health is oxidative stress, characterized by a disruption in the equilibrium between the production and accumulation of ROS within tissues, and the biological system’s efficacy in neutralizing or eliminating these reactive entities (Pizzino et al., 2017). Moreover, wound healing is intricately regulated by ROS, which play a pivotal role across various phases of the process. While low levels of ROS are essential for combating external damage, excessive oxidative stress combined with impaired antioxidant defenses, disrupts redox homeostasis and is a major contributor to the pathogenesis of nonhealing diabetic wounds (Deng et al., 2021). In the present study, oxidative stress-related parameters, including GSH, SOD, and MDA, were meticulously analyzed. Because of the known role of oxidative stress in wound healing, this study investigated the effect of empagliflozin on oxidative stress as it is a potential mechanism of action. One of the important antioxidants is GSH, which plays a pivotal role in the cellular defense system by counteracting oxidative damage and maintaining redox homeostasis. Elevated ROS levels necessitate both increased GSH content and metabolic resources to replenish its reserves (Ribeiro, 2023). Similarly, SOD catalyzes the rapid dismutation of superoxide anions, the primary ROS derived from molecular oxygen, into hydrogen peroxide to mitigate excessive oxidative reactions (Kurahashi and Fujii, 2015). Conversely, MDA, a well-established biomarker of oxidative stress, reflects elevated lipid peroxidation, a process primarily stimulated by excessive ROS activity (Bilgen et al., 2019).

The findings of the present study indicate that animals treated with empagliflozin (administered both orally and topically) demonstrate pronounced antioxidant activities, evidenced by elevated content of GSH and enhanced SOD activity, accompanied by a significant reduction in MDA concentration. These results corroborate prior findings demonstrating the antioxidative efficacy of empagliflozin in diabetic mice. In that study, empagliflozin (10 mg/kg/day via oral gavage for 8 weeks) not only ameliorated myocardial structural and functional abnormalities but also significantly reduced oxidative stress. In this previous study, the reduction in oxidative stress was achieved by downregulating NADPH oxidase 4 expression—a key NADPH oxidase isoform responsible for ROS production—while simultaneously enhancing myocardial antioxidant defenses. Improved glycemic control further contributed to the attenuation of oxidative injury, underscoring empagliflozin’s potential as a promising therapeutic agent for diabetic cardiomyopathy (Li et al., 2019). Regarding the topical application, to the best of our knowledge, this study represents the initial investigation into the antioxidant mechanisms underlying the topical use of empagliflozin.

The results of this study showed that MEBO^®^ demonstrated notable antioxidant efficacy, as evidenced by its ability to significantly modulate key oxidative stress markers. The treatment effectively enhanced GSH content and SOD activity, both of which are critical components of the antioxidant defense system. This suggests that MEBO^®^ supports cellular mechanisms to counteract oxidative stress, thereby preserving redox homeostasis. The results are consistent with previous findings in a study used an *in vivo* thermal burn model in male albino rats, where MEBO^®^ treatment increased both GSH content and SOD activity while reducing MDA. This effect may be partly due to β-sitosterol, a plant-derived steroid structurally similar to cholesterol and a component of MEBO^®^. By maintaining a moist environment, it promotes rapid re-epithelialization and tissue regeneration while inhibiting fungal and bacterial growth. Its strong anti-inflammatory, antioxidant, and antimicrobial properties reduce erythema and edema, supplying essential nutrients for faster healing (Khattab et al., 2024).

Furthermore, the study revealed that oral administration of empagliflozin and MEBO^®^ exhibited comparable antioxidant efficacy, as both treatments demonstrated similar modulation of antioxidant biomarkers (GSH and SOD) and the oxidative stress marker (MDA). Notably, both treatments exhibited significantly superior antioxidant effects compared to the topical application of empagliflozin, highlighting their enhanced ability to mitigate oxidative stress.

Inflammation, triggered by harmful stimuli such as infections or injury, induces leukocytes to release pro-inflammatory cytokines, such as IL-6 and TNF-α, which amplify inflammation by activating specific receptors, like the IL-6 receptor and the TNF-α receptor (Kiss, 2021). In wound healing, prolonged inflammation disrupts the transition to proliferation, fostering chronic wounds. Neutrophils and macrophages clear debris and pathogens, but impaired clearance or apoptosis perpetuates inflammation and delays healing (Holzer-Geissler et al., 2022). Given the central role of inflammation in wound healing, this study investigated the effect of empagliflozin on inflammation as a potential mechanism of action. The current results indicate that oral administration of empagliflozin markedly reduced inflammation, as evidenced by decreased concentrations of TNF-α and IL-6, compared to the untreated group. This result aligns with a previous clinical study involving 95 patients with DM and coronary artery disease, which demonstrated empagliflozin’s significant anti-inflammatory effects, evidenced by lower IL-6 concentration in patients treated with oral empagliflozin (Gohari et al., 2022). Additionally, another reported preclinical study confirmed the anti-inflammatory potential of oral empagliflozin is evident in its ability to reduce systemic and renal inflammation, effectively mitigating acute renal injury and lowering mortality in a mouse model of lipopolysaccharide-induced septic shock (Maayah et al., 2021). These studies reinforce the role of empagliflozin in modulating inflammation, which is critical for facilitating wound healing by resolving prolonged inflammatory phases.

By contrast, empagliflozin hydrogel and MEBO^®^ exhibited similar anti-inflammatory effects, though they were less substantial than those achieved with oral empagliflozin, as evidenced by TNF-α concentration. However, IL-6 concentrations were comparably reduced across all three treatments, indicating similar efficacy in this specific inflammatory marker. The study’s outcomes are in concordance with prior investigations exploring the repurposing of empagliflozin for psoriatic conditions. In an *in vivo* study of imiquimod-induced psoriasis in mice, empagliflozin gel at concentration of 1% and 3% was applied topically once daily for 7 days. The data revealed that the application of the gel significantly improved psoriasis. Furthermore, there was a concomitant decrease in the inflammatory biomarker TNF-α, with the 3% formulation demonstrating a more pronounced, dose-dependent reduction in TNF-α concentration. These findings suggest that topical empagliflozin may exert substantial anti-inflammatory and anti-psoriatic effects (Abbas et al., 2024).

STAT3 plays a critical role in wound healing by governing collagen deposition, which forming a structural framework vital for subsequent tissue regeneration. Moreover, STAT3 regulates the inflammatory cascade by modulating downstream mediators in a precisely controlled manner. This orchestrated inflammatory response is essential for pathogen elimination and wound debridement, ensuring an optimal environment for the initial phases of tissue repair. However, excessive STAT3 activation exacerbates fibrotic scar formation (Deng et al., 2024). The findings of the present study highlighted that both oral and topical administration of empagliflozin yielded the highest STAT3 concentrations. These findings are consistent with a preclinical experimental study, which revealed that oral empagliflozin enhances myocardial function, limits infarct size, and reestablishes redox equilibrium via STAT3- dependent antioxidant and anti-inflammatory pathways in a mice ischemia/reperfusion model. Empagliflozin-induced cardioprotection correlated with STAT3 upregulation, accompanied by diminished MDA concentration and suppressed myocardial inducible nitric oxide synthase (iNOS) and IL-6 expression (Andreadou et al., 2017). In contrast, MEBO^®^ and plain hydrogel exhibited STAT3 concentration approaching those observed in the untreated group. This suggests that MEBO^®^ and plain hydrogel lack the ability to significantly upregulate STAT3 expression. While these treatments may still contribute to wound healing through mechanisms independent of STAT3 activation. This highlights the unique role of empagliflozin in driving STAT3-mediated processes, which may explain its superior efficacy in enhancing tissue repair and protection compared to MEBO^®^ and plain hydrogel. To the extent of our knowledge, no previous studies have identified the STAT3 signaling pathway as a mechanistic target for the pharmacological actions of MEBO^®^.

Examining the distinct molecular mechanisms, oxidative stress converges on the activation of the Akt/Nrf2 signaling axis, albeit through divergent upstream triggers yet yielding overlapping downstream outcomes. This pathway plays a pivotal role in orchestrating endothelial cell survival, facilitating angiogenic processes, and preserving redox homeostasis (Abid et al., 2004; Zhao Y. et al., 2021). The activation of Akt via PI3K attenuates oxidative stress, concurrently suppressing autophagic and apoptotic processes. This intricate signaling cascade not only augments cellular resilience but also orchestrates and potentiates the activation of Nrf2, a key transcription factor regulating cellular antioxidant defenses (Xu et al., 2022). Upon activation by diverse stimuli, PI3K facilitates the phosphorylation and subsequent activation of Akt through PDK1. Activated Akt, in turn, orchestrates the activation of Nrf2 by modulating its nuclear translocation and stability, thereby inducing the transcription of Nrf2-regulated antioxidant and phase-II detoxification enzymes critical for cellular redox homeostasis. An alternative mechanism for Nrf2 activation involves the suppression of its negative regulator, GSK-3β, which constrains Nrf2 functionality by facilitating its degradation. Empirical evidence suggests that GSK-3β inhibition confers cytoprotective effects by directly modulating the Nrf2 signaling axis, fostering its stabilization, and augmenting antioxidant defense mechanisms (Wang et al., 2021). In skin, the PI3K/Akt pathway plays a critical role in maintaining epidermal barrier function, promoting hair follicle regeneration, facilitating wound healing, and counteracting skin senescence, while its activation safeguards melanocytes from apoptosis driven by oxidative stress (Teng et al., 2021). On the other hand, the pivotal role of Nrf2 in wound healing lies in its ability to detect elevated levels of ROS in damaged and inflamed tissues, thereby orchestrating the activation of antioxidant defense pathways to alleviate oxidative stress and facilitate tissue regeneration (Süntar et al., 2021). Therefore, this study investigates the effect of empagliflozin on these pathways as a potential mechanism contributing to its observed therapeutic effects.

With respect to the relative quantity of Akt, the current study demonstrated that oral empagliflozin significantly increased its relative abundance of Akt compared to the untreated and plain hydrogel groups. While topical empagliflozin also elevated Akt relative quantity, the increase was not statistically significant compared to the untreated or hydrogel groups. These findings highlight the superior efficacy of systematic oral empagliflozin in activating the PI3K/Akt signaling pathway, which is critical for promoting cell survival, angiogenesis, and redox homeostasis. Interestingly, the effect of oral empagliflozin on Akt activation may not be entirely related to its glucose-lowering ability. This is supported by a pre-clinical study in a left ventricular pressure overload model in mice induced by transverse aortic constriction, where empagliflozin improved systolic dysfunction by activating the Akt/eNOS/nitric oxide signaling pathway. This activation suppressed endothelial apoptosis, mitigated capillary rarefaction, and enhanced capillarization, effectively alleviating cardiac dysfunction (Nakao et al., 2021). These findings underscore empagliflozin’s potential to modulate Akt-related pathways, contributing to its therapeutic effects in wound healing and tissue repair.

On the other hand, MEBO^®^ treatment also increased Akt relative quantity, comparable to oral empagliflozin. The results align with a pre-clinical study on Wistar rats, revealing that MEBO^®^ promotes diabetic wound healing by enhancing autophagy and activating the PI3K/Akt/mammalian target of rapamycin pathway, as evidenced by increased autophagosomes and elevated PI3K and Akt expression (Zheng et al., 2020).

Regarding Nrf2 concentration, the present study revealed that topical empagliflozin showed no significant difference compared to the plain hydrogel group. However, oral systemic administration of empagliflozin significantly elevated Nrf2 concentration compared to the untreated group. Interestingly, this result does not appear to be related to empagliflozin’s ability to control blood glucose levels. Previous research demonstrated that oral empagliflozin exhibits significant antioxidant properties by directly activating the Nrf2/heme oxygenase-1 signaling pathway, independent of glucose-lowering effects. This was evident in a study evaluating the impact of empagliflozin in a non-diabetic model of bleomycin-induced pulmonary fibrosis in mice, where empagliflozin mitigated oxidative stress and attenuated fibrosis in pulmonary tissues (Kabel et al., 2020). Furthermore, the ability of oral empagliflozin to significantly elevate Nrf2 concentration appears to be unique to oral empagliflozin, as this effect was not observed with MEBO^®^, which showed comparable Nrf2 concentration as hydrogel in this study.

Integrating molecular pathways reveals a pivotal connection between Nrf2 and SIRT1, where enhanced nuclear translocation of Nrf2 suppresses p53 expression, a negative regulator of SIRT1, subsequently promoting the transcriptional activation of SIRT1. This regulatory interplay highlights a fundamental axis essential for sustaining cellular homeostasis and orchestrating stress response mechanisms (Yoon et al., 2016). Moreover, SIRT1 isoform of sirtuins plays a pivotal role in modulating Akt activation. SIRT1 facilitates the deacetylation of Akt, a critical modification that enhances its affinity for PIP3, thereby enabling its subsequent activation (Pillai et al., 2014). SIRT1 acts as an NAD-dependent deacetylase, catalyzing the removal of acetyl groups from a diverse array of protein substrates (Rahman and Islam, 2011). It plays a key role in sprouting angiogenesis and vascular growth by regulating endothelial cell proliferation, differentiation, and migration under hypoxic conditions (Pillai et al., 2014). Within the framework of diabetic wound healing, SIRT1 serves as a critical regulator, orchestrating a range of interconnected processes such as the attenuation of inflammatory pathways, enhancement of cellular migratory capacity, modulation of oxidative stress responses, and facilitation of granulation tissue development at the site of injury (Prabhakar et al., 2020). In this study, the effect of empagliflozin on SIRT1 was assessed, providing insights into its potential role in modulating these interconnected pathways and enhancing diabetic wound healing.

The outcomes of the current study revealed that both topical and oral empagliflozin elicited a significantly elevated concentration of SIRT1 compared to all other groups. These finding highlights that the elevation of SIRT1 is a unique marker for empagliflozin treatment. Importantly, this effect appears to be independent of empagliflozin’s ability to control blood glucose levels. Previous research on non-diabetic mice with experimentally induced preeclampsia involved administering angiotensin II type 1 receptor autoantibodies on gestation day 13, followed by daily oral treatment with empagliflozin until gestational day 19. This investigation assessed empagliflozin’s therapeutic effects on hypertension, proteinuria, and kidney injury, with specific emphasis on SIRT1 activation. This study highlights empagliflozin’s broader therapeutic potential beyond its role in glycemic control, demonstrating a significant attenuation of systolic hypertension and proteinuria, while simultaneously mitigating renal injury without compromising fetal outcomes. Additionally, a subset of the maternal mice was challenged with adriamycin at 12 weeks postpartum, and the results demonstrated that empagliflozin reduced adriamycin-induced kidney and podocyte injury in the postpartum period. These protective effects were mediated through SIRT1 activation (Zhai et al., 2022).

The Wnt/β-catenin signaling pathway plays a pivotal role across all stages of wound healing, with a particularly prominent function during the proliferative phase, where it orchestrates the formation of granulation tissue essential for repair (Gumede et al., 2024). The observations from the current study indicate that empagliflozin administration, via both oral and topical routes, significantly elevated β-catenin concentration *versus* the untreated group. Notably, the oral route of empagliflozin demonstrated greater efficacy compared to its topical application. Importantly, the effect of empagliflozin on β-catenin appears to be unique to this drug, as it was not observed in other groups. Furthermore, this effect is isolated from oral empagliflozin’s impact on hyperglycemia, as previous research on non-diabetic mice has demonstrated a similar result. This outcome aligns with findings from an *in vivo* study exploring the impact of empagliflozin in a mice model of cardiorenal syndrome type-3, defined by cardiac dysfunction secondary to acute kidney injury. Cardiomyocyte-specific FUN14 domain-containing protein 1 (FUNDC1) knockout mice underwent cardiorenal syndrome type-3 induction to assess mitochondrial function with or without empagliflozin. Empagliflozin maintained mitochondrial stability, reduced oxidative stress, and enhanced respiratory complex activity. It also activated β-catenin, promoted its nuclear localization, and increased FUNDC1-dependent mitophagy in cardiac tissue (Cai et al., 2022).

Interestingly, the therapeutic efficacy of oral empagliflozin in disease states not primarily related to glycemic control has been examined in clinical trials involving non-diabetic patients. The improvement in clinical outcomes with empagliflozin likely stems from mechanisms extending beyond glycemic regulation. In a double-blind, placebo- controlled trial, nondiabetic patients with heart failure with reduced ejection fraction (n = 84) were randomized to receive empagliflozin (10 mg daily) or placebo for 6 months. Empagliflozin administration in this cohort resulted in significant enhancements in left ventricular volumes, systolic function, and overall quality of life compared to placebo. These findings strongly reinforce the therapeutic role of empagliflozin in heart failure management, independent of glycemic status (Santos-Gallego et al., 2021). Another study validated empagliflozin’s efficacy beyond glycemic control. In a double-blind, placebo-controlled, single-center trial, 53 non-diabetic adults with a history of calcium or uric acid kidney stones were randomized to receive once-daily empagliflozin 25 mg for 2 weeks, followed by placebo, or *vice versa*. Empagliflozin significantly improved the urinary lithogenic risk profile, endorsing it as a safe strategy to prevent kidney stone recurrence (Anderegg et al., 2025).

**FIGURE 9:**
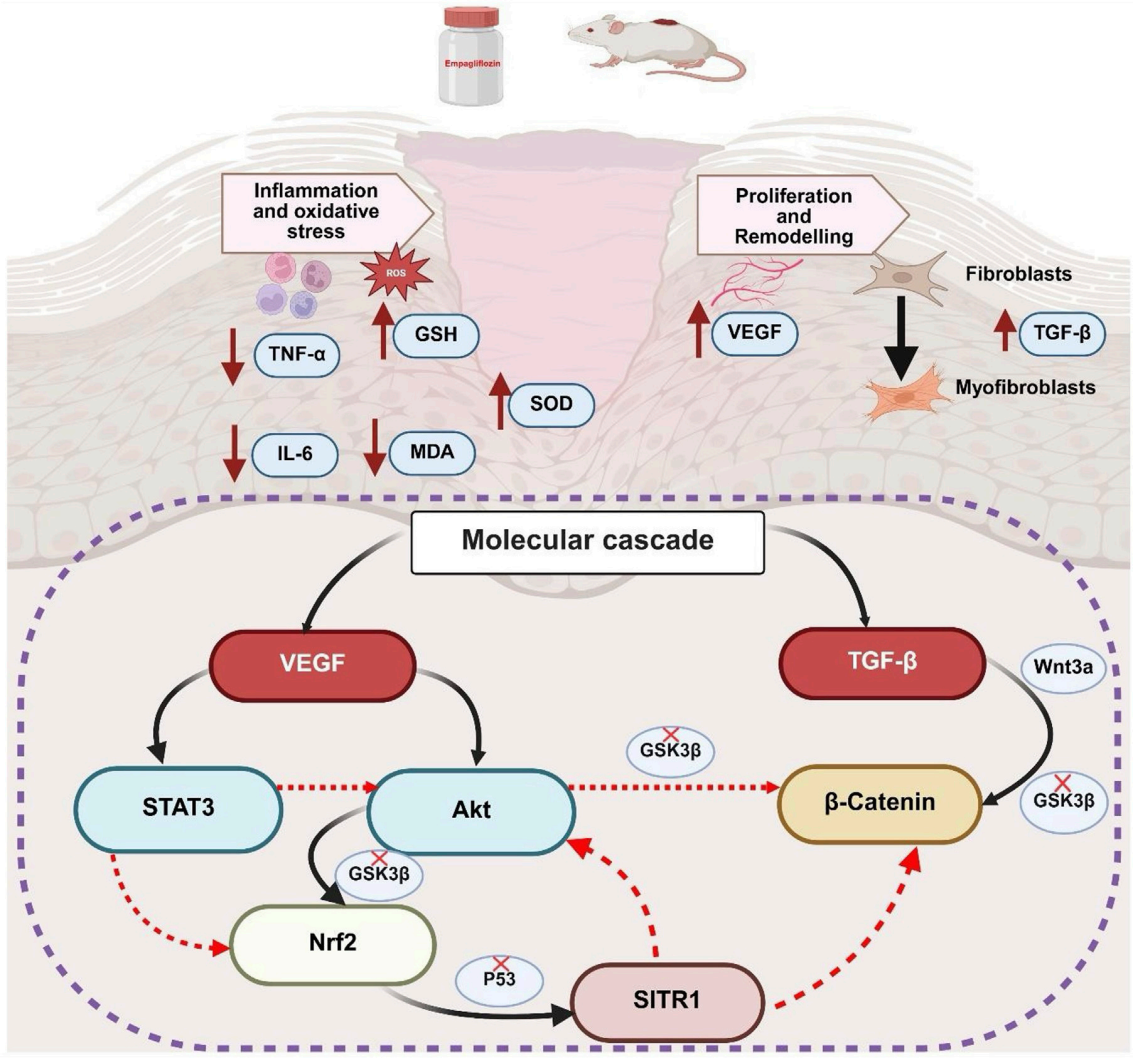
Summary of mechanistic and integrated molecular pathways of empagliflozin. GSH, reduced glutathione; SOD, superoxide dismutase; MDA, malondialdehyde; TNF-α, tumor necrosis factor-alpha; IL-6, interleukin-6; VEGFA, vascular endothelial growth factor A; TGF-β1, transforming growth factor beta 1; STAT3, signal transducer and activator of transcription 3; Akt, protein kinase B; Nrf2, nuclear factor erythroid 2-related factor 2; SIRT1, sirtuin 1; GSK3β, glycogen synthase kinase-3 beta; Wnt3a, wingless-related integration site 3 a.

In summary, both topical and oral administration of empagliflozin demonstrates potential efficacy in promoting wound healing, albeit with distinct mechanisms of action. The enhanced wound-healing effects observed with oral empagliflozin are likely attributable to its dual role in glycemic control and anti-inflammatory and antioxidant properties. However, empagliflozin hydrogel demonstrated a significant improvement in wound healing despite having no effect on FBG levels, suggesting that its mechanism of action may involve pathways beyond glycemic control. These effects are mediated through indirectly activation of key molecular pathways pivotal to tissue repair, such as STAT3, Akt, Nrf2, SIRT1, and β-catenin as summarized in Figure 9. Notably, while oral administration showed superior outcomes across several parameters, topical empagliflozin, despite lacking glycemic effects, facilitated wound healing through similar molecular mechanisms. Collectively, these findings highlight the complementary therapeutic potential of oral and topical empagliflozin to develop a comprehensive and effective therapeutic approach to enhance wound healing.

Based on the findings of the current study, further experimental investigations are necessary to validate these results and to uncover the precise molecular mechanisms by which empagliflozin influences wound healing, particularly focusing on pathways such as Notch and MAPK. Additionally, future research should assess the incidence of adverse effects associated with empagliflozin at varying dosages and over extended treatment durations to provide a thorough evaluation of its safety profile. Clinical studies are also recommended to explore the therapeutic efficacy of empagliflozin in diabetic patients with impaired wound healing, with the aim of determining its potential benefits in promoting tissue repair and recovery.

## Data Availability

The original contributions presented in the study are included in the article/supplementary material, further inquiries can be directed to the corresponding author.
